# Mechanisms and Therapeutic Potential of Myofibroblast Transformation in Pulmonary Fibrosis

**DOI:** 10.70322/jrbtm.2025.10001

**Published:** 2025-03-07

**Authors:** Tianming Zhao, Yunchao Su

**Affiliations:** 1 Department of Pharmacology & Toxicology, Medical College of Georgia at Augusta University, Augusta, GA 30912, USA; 2 Division of Pulmonary and Critical Care Medicine, Department of Medicine, Medical College of Georgia at Augusta University, Augusta, GA 30912, USA; 3 Vascular Biology Center, Medical College of Georgia at Augusta University, Augusta, GA 30912, USA; 4 Research Service, Charlie Norwood Veterans Affairs Medical Center, Augusta, GA 30912, USA

**Keywords:** Pulmonary fibrosis, Extracellular matrix, Fibroblasts, Myofibroblasts, Cytokines

## Abstract

Idiopathic pulmonary fibrosis (IPF) is a progressive, irreversible, and fatal disease with an increasing incidence and limited therapeutic options. It is characterized by the formation and deposition of excess extracellular matrix proteins resulting in the gradual replacement of normal lung architecture by fibrous tissue. The cellular and molecular mechanism of IPF has not been fully understood. A hallmark in IPF is pulmonary fibroblast to myofibroblast transformation (FMT). During excessive lung repair upon exposure to harmful stimuli, lung fibroblasts transform into myofibroblasts under stimulation of cytokines, chemokines, and vesicles from various cells. These mediators interact with lung fibroblasts, initiating multiple signaling cascades, such as TGFβ1, MAPK, Wnt/β-catenin, NF-κB, AMPK, endoplasmic reticulum stress, and autophagy, contributing to lung FMT. Furthermore, single-cell transcriptomic analysis has revealed significant heterogeneity among lung myofibroblasts, which arise from various cell types and are adapted to the altered microenvironment during pathological lung repair. This review provides an overview of recent research on the origins of lung myofibroblasts and the molecular pathways driving their formation, with a focus on the interactions between lung fibroblasts and epithelial cells, endothelial cells, and macrophages in the context of lung fibrosis. Based on these molecular insights, targeting the lung FMT could offer promising avenues for the treatment of IPF.

## 1. Introduction

Pulmonary fibrosis is a chronic irreversible interstitial lung disease characterized by excessive scarring of the lungs. Following exposure to damaging agents, lung tissue experiences abnormal repair processes, where scar tissue gradually replaces the healthy lung architecture. This replacement impairs gas exchange, reduces lung function, and frequently leads to fatal outcomes [1]. Pulmonary fibrosis can be triggered by identifiable harmful agents, including pathogens such as the severe acute respiratory syndrome coronavirus 2 (SARS-CoV-2), silica dust, radiation, certain medications, and some autoimmune disorders [2–6]. When pulmonary fibrosis arises without any identifiable cause, it is termed idiopathic pulmonary fibrosis (IPF). There are no effective treatment options yet for IPF, because the exact mechanisms underlying pulmonary fibrosis are not fully understood [7]. It is believed that repeated injuries to lung tissue lead to the release of proinflammatory mediators, which in turn activate immune cells and intensify the inflammatory response [8,9]. Subsequently, pulmonary fibroblasts are activated and orchestrate with epithelial, immune, and endothelial cells (ECs) through immunomodulatory and angiogenic signaling pathways [10]. Under normal conditions, this initial response is followed by fibroblast apoptosis and the removal of excess collagen, which helps restore normal tissue architecture [11,12]. Under persistent and repeated injuries, fibroblasts or other lung cells are driven to differentiate into contractile myofibroblasts that secrete excessive amounts of extracellular matrix (ECM) proteins. More importantly, differentiated myofibroblasts characteristically express alpha-smooth muscle actin (α-SMA), which contributes to stronger contractility in pulmonary fibrosis [13,14].

In the context of pulmonary fibrosis, fibroblasts and myofibroblasts, originating from various cell types, proliferate and accumulate in the lung interstitium, leading to the formation of fibro-foci and compromising the integrity of lung structure. Myofibroblasts are central to the excessive deposition of ECM and are activated in response to various pathological conditions. They are also key targets for antifibrotic therapies. This review highlights recent advances in understanding the roles of fibroblast to myofibroblast transformation (FMT) in pulmonary fibrosis, including their characteristics and the exogenous and endogenous factors that regulate FMT. Additionally, we will discuss novel antifibrotic drugs currently in development targeting FMT in pulmonary fibrosis.

## 2. Origins of Lung Myofibroblasts

**Figure 1.**
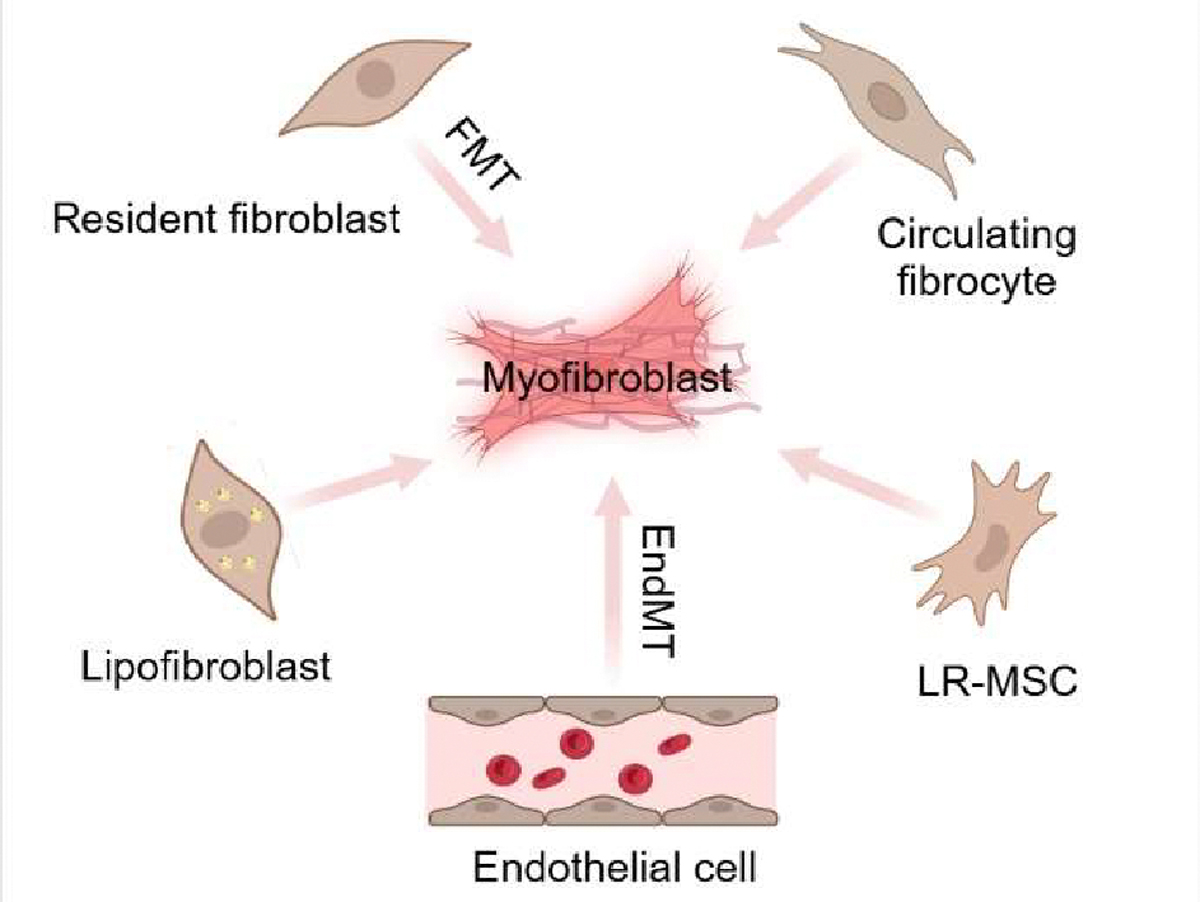
Myofibroblasts arise from different precursor cells. Myofibroblasts are derived from multiple sources, including resident fibroblasts, circulating fibrocytes, lipofibroblasts, lung resident mesenchymal stem cells, and vascular ECs.

Lung myofibroblasts can arise from a variety of cell types under both normal and pathological conditions. It is suggested that the myofibroblast population is heterogeneous, with cells deriving from multiple sources, including resident fibroblasts, circulating fibrocytes, lipofibroblasts, lung resident mesenchymal stem cells, and vascular ECs (Figure 1).

### From resident fibroblasts:

Pulmonary resident fibroblasts, which originate from the mesoderm, play a vital role in the formation and elongation of alveolar septa after birth. These fibroblasts remain in a quiescent state until activated by external stimuli. Cell-fate mapping confirmed that alveolar fibroblasts which constitute the majority of LEPR+ fibroblasts, differentiate into pathological fibroblasts in bleomycin-induced pulmonary fibrosis and serve as a key source of myofibroblasts [15]. Various factors, including cytokines such as interleukin (IL)-1β, IL-6, tumor necrosis factor (TNF), and transforming growth factor beta (TGFβ) and chemokines, can induce the transformation of these cells into myofibroblasts [16–19]. Among these factors, TGFβ1 is recognized as a key mediator in driving FMT [20,21]. Beyond the classical TGFβ signaling pathway, which involves the phosphorylation of SMAD family member 2 (SMAD2) and SMAD3, non-canonical TGFβ pathways such as those activating extracellular signal-regulated kinase (ERK), c-Jun N-terminal kinase (JNK), and p38 mitogen-activated protein kinase (p38), are also implicated in lung FMT [22–25].

### From circulating fibrocytes:

In response to tissue injury, circulating fibrocytes enter the peripheral bloodstream from the bone marrow, and are then recruited to the injury site for tissue repair [26]. These cells express CD45, collagen I, and CXC chemokine receptor 4 (CXCR4). They migrate into the lung interstitium in response to CXC chemokine ligand 12 (CXCL12), are differentiated to myofibroblasts in pulmonary fibrosis [27]. The CXCR4 inhibitor AMD3100, which is clinically used for mobilizing hematopoietic stem cells [28], has been shown to alleviate pulmonary fibrosis following bleomycin exposure [29]. In a mouse model of silicosis, AMD3100 also reduced the influx of circulating fibrocytes into the lung, resulting in a delayed onset of pulmonary inflammation and fibrosis [30]. The proportion of circulating fibrocytes is elevated in patients with IPF [31]. Circulating fibrocytes from elderly individuals exhibit higher levels of IL-18 and its receptor, which enhances their ability to differentiate into myofibroblasts [32]. This may partially explain the age-related increase in pulmonary fibrosis. Elevated circulating fibrocyte levels are associated with increased overall mortality in a large IPF cohort [33]. Additionally, blocking the mammalian target of rapamycin (mTOR) pathway with sirolimus has been shown to reduce circulating fibrocytes in both IPF patients and in a bleomycin-induced pulmonary fibrosis model, and it also mitigates collagen deposition in the lung tissues of mice [34,35].

### From lipofibroblasts:

Lipofibroblasts are a special type of fibroblast characterized by the presence of lipid droplets in the cytoplasm and the phenotypical expression of adipose differentiation-related protein (ADRP) and perilipin2 (PLIN2) as well as peroxisome proliferator-activated receptor gamma (PPARγ). These cells are anatomically located in proximity to alveolar epithelial type 2 (AT2) cells [36]. Lipofibroblasts significantly contribute to the expansion of the myofibroblast population in pulmonary fibrosis [36]. Recent studies have identified a phenotypic switch from lipofibroblasts to myofibroblasts during the initiation of fibrosis, with a reversal from myofibroblasts to lipofibroblasts during fibrosis resolution [37]. Single-cell RNA-Seq and lineage tracing revealed that bleomycin-injured lipofibroblasts acquire myogenic activity and lose adipogenic activity [38]. Metabolic reprogramming promotes the phenotypic transformation from lipofibroblasts to profibrotic myofibroblasts by downregulating the adipogenic transcription factors 21 (TCF21) and PPARγ in IPF [39]. FGF18/fibroblast growth factor receptor (FGFR) signaling facilitates the differentiation of mesenchymal progenitors by reducing the expression of myofibroblast markers (ACTA2, COL1A1) while increasing the expression of lipofibroblast markers (ADRP and PPARγ) [40].

### From lung resident mesenchymal stem cells:

Lung resident mesenchymal stem cells (LR-MSCs) reside in the lung’s interstitial spaces and perivascular regions. They can remain quiescent under normal conditions but are activated in response to lung injury or inflammation [41]. A lineage tracing approach identified TBX4-lineage mesenchymal progenitors as the major source of myofibroblasts in the injured adult lung [42]. Upon lung injury, LR-MSCs can differentiate into myofibroblasts, driven by fibrogenic factors such as TGFβ. This differentiation process is characterized by the acquisition of myofibroblast markers like α-SMA and the increased production of extracellular matrix components, including collagen and fibronectin [43–45]. LR-MSCs have emerged as a potential source of myofibroblasts in the context of pulmonary fibrosis.

### From vascular endothelial cells:

ECs make up about 30% of the cellular composition in the normal lung [46]. Pulmonary capillary ECs have been shown to differentiate to fibroblasts and myofibroblasts through the process of endothelial-mesenchymal transition (EndMT) [47–49]. The loss of pulmonary microvascular endothelial cells (PMVECs) and the disruption of regulated angiogenesis are crucial mechanisms in the development of pulmonary fibrosis [50,51]. In IPF, damage to epithelial cells triggers the release of active TGFβ, which subsequently activates ECs. This activation disrupts the balance of vasostatic and angiogenic mediators, leading to abnormal endothelial cell proliferation and apoptosis [52]. Cell lineage tracing reveals that cells originating from the endothelial lineage accounted for nearly 20% of the total cell population in fibrotic foci in the bleomycin model [53], contributing to pulmonary fibrosis [54]. It has been shown that COVID-19 infection promotes transition of ECs to EC-like myofibroblasts and myofibroblasts in COVID-19-related pulmonary fibrosis [55]. In pulmonary fibrosis tissues, EC-like myofibroblasts exhibit reduced expression of endothelial markers (VE-cadherin and von Willebrand factor) and increased expression of myofibroblast markers, including α-SMA, collagen type I alpha 1 (COL1A1), and fibronectin 1 (FN1), compared to healthy controls. Furthermore, endothelial cell-specific loss of matrix gla protein (MGP) leads to pulmonary fibrosis, with MGP deficiency in ECs promoting a greater transformation into EC-like myofibroblasts and myofibroblasts [54].

### From alveolar epithelial cells:

Epithelial-mesenchymal transition (EMT) represents a critical pathological process in the pathogenesis of pulmonary fibrosis [24]. It has been demonstrated that in vitro exposure of epithelial cells to TGFβ1 or bleomycin induces a downregulation of epithelial markers such as E-cadherin and cytokeratin, accompanied by an upregulation of mesenchymal markers, including N-cadherin, vimentin, and α-SMA [24,56]. Following EMT, epithelial cells acquire stromal cell-like characteristics and were hypothesized to contribute to the pool of lung fibroblasts in the context of pulmonary fibrosis [57]. However, animal studies employing cell lineage tracing of AT2 cells in the bleomycin-induced mouse model have shown that AT2 cells do not transit into fibroblasts or myofibroblasts and do not serve as a source of myofibroblasts through EMT [58]. Nevertheless, inhibiting EMT has been reported to attenuate the progression of pulmonary fibrosis [56,59].

### From pericytes:

Lung pericytes are mesenchyme-derived cells residing within the basement membranes or perivascular lining that play a critical role in collagen production in wound healing [60]. Pericytes differentiate into myofibroblasts through the process of pericyte-myofibroblast transformation (PMT) [61–63]. Human pericytes respond to profibrotic factors in the fibrotic lung microenvironment, generating a cell population with myofibroblast-like characteristics. Even a slight increase in substrate stiffness is sufficient to prime pericytes to adopt a myofibroblast-like phenotype via mechanotransduction signals [64]. In the bleomycin pulmonary fibrosis model, forkhead box D1 (Foxd1) progenitor-derived pericytes proliferate and transform into cells with α-SMA expression in fibrotic foci [65]. Notch1, which is upregulated in IPF lung tissue, has been shown to mediate lung pericyte proliferation and differentiation into myofibroblasts via the platelet-derived growth factor receptor β (PDGFRβ)/rho-associated protein kinase 1 (ROCK1) pathway [61]. Nevertheless, lineage tracing of pericyte-like cells in the alveolar interstitium using inducible transgenic alleles revealed heterogeneity among proliferating cell types in fibrotic lesions and failed to confirm pericytes as the primary origin of myofibroblasts [58], although they contribute to α-SMA+ cells that surround endothelial cells [15]. Therefore, further studies are needed to better understand the characteristics of pericytes and their transition to myofibroblasts under pathological conditions.

## 3. Properties of Myofibroblasts

### Proliferation and resistance to apoptosis:

In normal lung tissue, fibroblasts interact with polymerized type I collagen through the α2β1 integrin, which maintains phosphatase and tensin homolog (PTEN) activity and inhibits the phosphatidylinositol 3-kinase/protein kinase B (PI3K/AKT) proliferation signaling pathway. This interaction provides an effective physiological mechanism to prevent excessive fibroblast proliferation following tissue damage [66]. However, during fibrosis, profibrotic factors such as TGFβ1 drive the transformation of fibroblasts into myofibroblasts, which results in these cells acquiring a variety of behavioral differences, including vigorous proliferation potential, forceful migration, excessive contractility, and resistance to apoptosis [67–70]. When stimulated by TGFβ, human fibroblasts deposit abnormal α1 and α2 collagen IV chains, altering the composition of the basement membrane. This change hinders myofibroblast migration and promotes their survival, potentially contributing to the persistence and prolonged activity of myofibroblasts within fibroblastic foci [71–73].

Compared to normal fibroblasts, myofibroblasts derived from lung fibrosis exhibit resistance to FasL-induced apoptosis, showing lower levels of surface-bound Fas and higher levels of soluble Fas (sFas) [74]. Matrix metalloproteinases (MMPs) convert membrane-bound FasL into soluble FasL, which helps maintain resistance to FasL-mediated cell death in fibrotic lung myofibroblasts [75]. The loss of Fas signaling decreases fibroblast apoptosis and promotes persistent pulmonary fibrosis [76]. In myofibroblasts from fibroblastic foci in IPF patient specimens, both Fas and cleaved caspase-3 proteins are low. One possible mechanism is that low forkhead box O3 (FOXO3a) activity suppresses Fas expression through a PTEN/AKT-dependent pathway, thereby conferring an anti-apoptotic phenotype on myofibroblasts in IPF [77]. Additionally, fibrotic fibroblasts express high levels of Wnt family member 5A (WNT5A). When normal or usual interstitial pneumonia (UIP) lung fibroblasts are treated with WNT5A, their proliferation increases, and they exhibit greater resistance to H_2_O_2_-induced apoptosis [68]. Overall, the high proliferation and apoptosis resistance of myofibroblasts drive the excessive deposition of ECM in fibrosis [22,39,78].

### Plasticity:

Myofibroblasts typically return to a quiescent state and may undergo apoptosis or senescence to reduce their numbers after injury is repaired [79,80]. Myofibroblasts can also exhibit a broader spectrum of lineage plasticity, de-differentiate into lipofibroblasts and fibroblasts at various stages of tissue repair [81–83]. For example, in fibrotic lung tissue, myofibroblasts may cease their contractile activity and reprogram into novel adipogenic cells in response to bone morphogenetic protein (BMP) signaling [84]. This increase in lipofibroblasts is associated with a reduced capacity to synthesize collagen and an enhanced ability to degrade collagen in fibrotic foci. While the transformation from lipofibroblasts to myofibroblasts is dependent on TGFβ1, the de-differentiation from myofibroblasts to lipofibroblasts is likely mediated by PPARγ [37]. Omentin-1 significantly upregulates PPARγ, facilitating the dedifferentiation of myofibroblasts into a lipofibroblast phenotype during the resolution of pulmonary fibrosis [83]. Metformin has been shown to promote lipofibroblast formation in IPF lung tissue and help fibrosis resolution through enhancing PPARγ activity and expression that is independent of AMP-activated protein kinase (AMPK) signaling [84]. Additionally, myofibroblasts can de-differentiate into fibroblasts, restoring their sensitivity to apoptosis and contributing to the resolution of lung fibrosis [81,85]. Notably, these fibroblasts remain responsive to TGFβ1 and can be re-induced to differentiate into myofibroblasts [86].

### Myofibroblast markers:

Lung myofibroblasts have been shown to be heterogeneous in both patients and mouse models of pulmonary fibrosis, as they may originate from distinct cell populations, as previously mentioned. Myofibroblasts differentiated from various cell types exhibit distinct differences in gene expression [87]. Myofibroblasts are characterized by the expression of α-SMA (Acta2), which distinguishes them from fibroblasts. They exhibit increased synthesis of ECM components and display enhanced contractility [88]. The expression of α-SMA or COL1A1 is commonly recognized as a marker of myofibroblast transformation. In vitro, lung fibroblasts are typically induced to differentiate into myofibroblasts using TGFβ1, characterized by *de novo* expression of α-SMA. The presence of α-SMA within stress fibers enhances the contractile capacity of myofibroblasts [89]. In fibrotic lungs, only a small subset of collagen-producing cells co-expresses α-SMA [90], and α-SMA-expressing cells represent only a subset of PDGFRβ-expressing cells in the fibrotic lung interstitium [90].

Single-cell RNA sequencing (scRNA-seq) allows for a more detailed assessment of lung fibroblast and myofibroblast diversity, aiding in the identification of novel marker genes. ScRNA-seq revealed CD82 as a marker for lipofibroblasts and CD239 as a marker for myofibroblasts [91]. Additionally, CD82 was found to co-localize with PDGFα+ fibroblasts in IPF lung tissue [92]. Further scRNA-seq studies identified a subset of cells with high expression of collagen triple helix repeat containing 1 (CTHRC1) which is specific to cells with the highest collagen expression, while no significant expression of CTHRC1 was detected in other lung cell types. In contrast, the myofibroblast marker α-SMA was predominantly expressed by low collagen-expressing cells. Notably, α-SMA is not a specific marker for pathological fibroblasts that produce the highest levels of ECM. CTHRC1+ fibroblasts may contribute to pathological fibrogenesis in IPF [9].

In a bleomycin-induced mouse model, the use of established myofibroblast marker genes such as Acta2, Myh11, and Tagln led to the identification of three additional myofibroblast markers, Hhip, Aspn, and Mustn1, which proved to be more effective markers for myofibroblasts than α-SMA [93]. However, further validation of the specificity of these markers through histological analysis is still required.

## 4. Regulators of Myofibroblast Transformation

### TGFβ canonical signaling pathway:

TGFβs are key cytokines that promote the transformation of lung fibroblasts into myofibroblasts. TGFβs are dimeric proproteins composed of a C-terminal growth factor (mature TGFβ) and an N-terminal pro-peptide known as the latency-associated peptide (LAP). In the endoplasmic reticulum, TGFβ forms a complex with the latent TGFβ binding protein (LTBP) through disulfide bonds between LTBP and LAP. This complex is then transported to the Golgi apparatus, where it is cleaved by the endogenous protease furin to produce mature TGFβ [94,95]. Activated TGFβ forms a ligand-receptor complex with the TGFβ type II receptor (TGFβR2), which is subsequently recognized by the type I receptor (TGFβR1) [96]. Once activated, TGFβR1 induces the phosphorylation of SMAD2 and SMAD3, which then form a complex with SMAD4 and translocate to the nucleus to regulate the transcription of fibrosis-related target genes, including fibronectin, collagen, and connective tissue growth factor (CTGF) [97–99]. SMAD6 and SMAD7 function as inhibitory factors that suppress TGFβ signaling [100]. In addition to activating the canonical TGFβ/SMAD pathway, TGFβ can also activate non-canonical (non-SMAD) signaling pathways, including the p38 mitogen-activated protein kinases (p38 MAPK), the extracellular signal-regulated kinase (ERK), PI3K/AKT, and Janus kinase 1/signal transducer and activator of transcription 3 (JAK1/STAT3) pathways [101–103].

**Figure 2.**
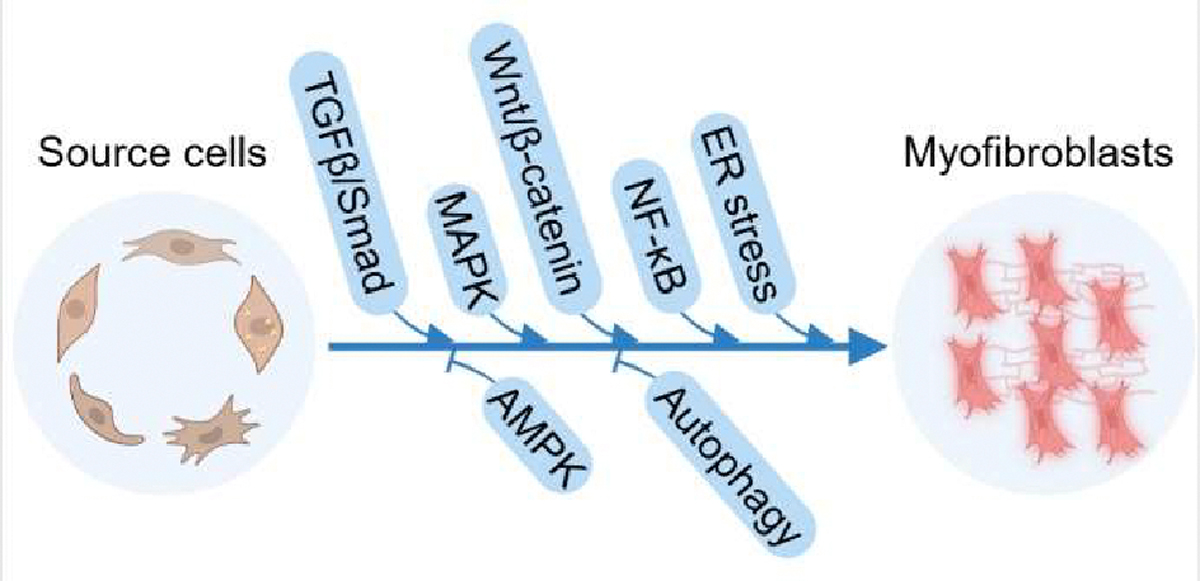
Signaling pathways associated with myofibroblast transformation. TGFβ/Smad, MAPK, Wnt/β-catenin, NF-κB and ER stress pathways promote myofibroblast transformation. AMPK and autophagy pathway inhibit myofibroblast transformation.

In the lungs, TGFβ is produced by various cell types, including alveolar macrophages, neutrophils, activated alveolar epithelial cells, ECs, fibroblasts, and myofibroblasts [104]. TGFβ, in turn, acts on multiple cell types such as epithelial cells, macrophages, and ECs, with fibroblasts being the primary target [94]. In cell culture, TGFβ1 induces lung fibroblasts to express α-SMA and to produce various ECM components and to synthesize the enzymes and chaperones necessary for ECM assembly, leading to FMT [105]. Biologically active TGFβ1 has been shown to drive pulmonary myofibroblast differentiation through both autocrine and paracrine signaling (Figure 2). All TGFβ isoforms and receptors are reported to be upregulated and activated in IPF, with TGFβ1 playing a pivotal role in its pathogenesis [106]. Adenovirus-mediated transfer of the active TGFβ1 gene results in long-term severe pulmonary interstitial and pleural fibrosis, characterized by extensive deposition of ECM proteins such as collagen, fibronectin, and elastin, along with the emergence of cells with a myofibroblastic phenotype [107]. TGFβ1 expression is also elevated in bleomycin-induced fibrotic lung tissue [108].

TGFβ1-induced transformation of lung myofibroblasts is associated with altered gene expression, which can further regulate TGFβ1 function. For instance, exposure to TGFβ leads to the upregulation of programmed cell death 1 ligand 1 (PD-L1) via the SMAD3 and p38 pathways. Knockdown of PD-L1 significantly reduces TGFβ-induced myofibroblast activation in both normal and IPF lung fibroblasts [109,110]. Additionally, TGFβ stimulates the incorporation of lung fibroblast-derived PD-L1 into extracellular vesicles (EVs), which can inhibit T cell proliferation and facilitate fibroblast migration [110]. PD-L1 mediates the invasive behavior of lung fibroblasts and promotes lung fibrosis in a humanized mouse model of IPF. Targeting PD-L1 through knockout strategies, focal adhesion kinase (FAK) inhibitors, or PD-L1 neutralizing antibodies reduces invasion and attenuates fibrosis, suggesting that PD-L1 could be a novel therapeutic target in IPF [111]. Moreover, TGFβ1 suppresses the expression of the transcription factor forkhead box F1 (FOXF1) in lung fibroblasts. Activation of FOXF1 can inhibit FMT and slow the progression of silicotic fibrosis by repressing the canonical TGFβ/SMAD2/3 pathway [112].

### MAPK signaling pathway:

The MAPK pathway comprises three major signaling cascades: ERK, JNK, and p38 MAPK. These cascades are activated by a variety of extracellular stimuli, including growth factors, cytokines, and stress signals, leading to a series of phosphorylation events that ultimately regulate gene expression and cellular processes such as proliferation, differentiation, apoptosis, and stress responses [113]. TGFβ can activate MAPK pathway that represents non-canonical (non-SMAD) signaling, which contributes to the synergistic enhancement of FMT and ECM production [101–103]. Overexpression of FRNK blocks the activation of the MAPK pathway and thus inhibits the upregulation of α-SMA expression induced by TGFβ1 [114].

The ERK pathway is particularly important in lung myofibroblast transformation (Figure 2). Lung FMT, proliferation, and migration in silica exposure-induced pulmonary fibrosis is through the ERK/activator protein-1 (AP-1)/nucleophosmin (NPM1) signaling pathway [115]. IL-11 stimulates lung FMT in an ERK-dependent autocrine manner [116]. Vincristine, an anti-tumor drug that induces pulmonary fibrosis as a side effect, promotes the production of collagen, vimentin, and α-SMA in embryonic lung fibroblasts, accompanied by the phosphorylation of ERK and p38 effects [117]. Similarly, the antiarrhythmic drug amiodarone promotes the FMT and proliferation via the phosphorylation of ERK and p38 [25].

JNK is a stress-activated protein kinase with multiple isoforms (JNK1, JNK2, and JNK3). The JNK pathway is activated by stress stimuli such as DNA damage, cytokines, and oxidative stress [118]. Inhibition of JNK blocks FMT. FGF19 has been shown to prevent TGFβ-induced myofibroblast differentiation by inhibiting JNK phosphorylation [119]. CC-90001 inhibits TGFβ1-induced α-SMA synthesis in lung fibroblasts [120] and has completed phase I and II clinical trials, showing improvements in lung function [120–122].

The p38 MAPK can be activated by various stress signals, including pro-inflammatory cytokines and environmental stressors [123]. In pulmonary fibrosis, p38 MAPK activation leads to FMT and increased production of collagen and other ECM components [25,117]. MAPK phosphatase inhibition of p38α within lung myofibroblasts is essential for spontaneous fibrosis resolution [81].

### AMPK signaling pathway:

In pulmonary fibrosis, AMPK serves as a potent inhibitor of FMT (Figure 2). Metformin-induced AMPK signaling has been shown to attenuate TGFβ-induced myofibroblast differentiation in human lung fibroblasts, significantly reducing phosphorylated STAT3 levels and inhibiting FMT in bleomycin-induced pulmonary fibrosis in mice [84,124,125]. TGFβ1 has been reported to inhibit AMPK phosphorylation in mouse primary lung fibroblasts [59]. Numerous studies have demonstrated the therapeutic potential of AMPK activation in animal models of pulmonary fibrosis [126–128]. In addition, TGFβ-induced lung FMT is associated with a reduction of autophagy caused by activated mTOR pathway [129]. AMPK signaling can inhibit mTOR function, thereby enhancing autophagy [130,131]. However, it remains to be explored whether AMPK inhibits lung FMT by promoting autophagy.

### Wnt/β-catenin signaling pathways:

In both human IPF and experimental lung fibrosis, Wnt signaling is aberrantly active, as evidenced by the dysregulated expression of various Wnt ligands and receptors [132–134]. For example, Wnt8b is upregulated in myofibroblasts derived from LR-MSCs treated with TGFβ1, as well as in lungs of bleomycin-induced fibrosis. Wnt8b regulates the differentiation of LR-MSCs into myofibroblasts through the Wnt/β-catenin signaling pathway [135]. The Shh/Gli signaling promotes the transformation of LR-MSCs into myofibroblasts by upregulating Wnt10a expression during pulmonary fibrosis [133]. TRAF6 inhibits fibroblast activation by reducing Wnt3a expression [136]. Activation of Wnt/β-catenin signaling is required for TGFβ1-induced myofibroblast transformation [137,138]. WNT5A regulates the activation of latent TGFβ, driving myofibroblast transformation and contributing to lung fibrosis [139].

### NF-κB signaling pathway:

Beyond its well-known role in inflammation, NF-κB has been implicated in myofibroblast transformation (Figure 2). TNF-α-induced NF-κB activation drives the differentiation of lung resident mesenchymal stem cells (LR-MSCs) into myofibroblasts, exacerbating bleomycin-induced pulmonary fibrosis [140]. Poly(I) enhances NF-κB translocation into the nucleus, promoting the expression of TGFβ1 and α-SMA in human fetal lung fibroblasts [141]. High-mobility group box 1 (HMGB1) is another proinflammatory factor that induces fibroblast-to-myofibroblast differentiation through NF-κB. Inhibition of NF-κB in HMGB1-stimulated cells reduces TGFβ1 release and myofibroblast marker expression [142]. LTBP2 (latent transforming growth factor β binding protein 2) also regulates fibroblast-to-myofibroblast differentiation by activating NF-κB signaling [143]. Additionally, SIRT6 interacts with the NF-κB subunit p65 and inhibits TGFβ1-induced NF-κB-dependent transcriptional activity, thereby preventing TGFβ1-induced lung FMT [144]. Inhibiting NF-κB phosphorylation and nuclear translocation effectively prevents lung FMT and exerts anti-fibrotic effects in vivo [143,145–147].

### Autophagy:

In pulmonary fibrosis, levels of p62 and ubiquitinated proteins are elevated in epithelial cells and fibroblasts within fibrotic lesions, compared to relatively normal areas, suggesting a deficiency in autophagy activity [129,148]. Inadequate autophagy accelerates epithelial cell senescence, promotes ECM deposition and FMT in lung fibroblasts, and speeds up the fibrotic process [148–151]. TGFβ1 has been shown to inhibit autophagy in fibroblasts [129]. However, another study demonstrated that TGFβ1 induces autophagy in both non-IPF and IPF fibroblasts, with autophagy contributing to the profibrotic effects of TGFβ1 [152]. The potential reason for these different effects is that fibroblasts treated with TGFβ1 for an extended period may exhibit varied responses. Despite this, increasing evidence suggests that inducing or restoring autophagy has anti-fibrotic effects [153–155]. Further studies are necessary to explore the regulatory network of autophagy in pulmonary fibrosis.

### Endoplasmic reticulum stress:

The endoplasmic reticulum (ER) is a vital cellular organelle responsible for protein folding, lipid synthesis, and calcium storage [156]. Under normal physiological conditions, the ER ensures proper protein folding and maturation. However, various pathological conditions can disrupt ER homeostasis, leading to the accumulation of misfolded or unfolded proteins within the ER lumen, a condition known as ER stress [156,157]. ER stress triggers the activation of the unfolded protein response (UPR), a signaling network designed to restore ER homeostasis. The UPR consists of three main branches, mediated by inositol-requiring enzyme 1 (IRE1), activating transcription factor 6 (ATF6), and protein kinase RNA-like ER kinase (PERK) [156,157]. However, persistent ER stress and prolonged UPR activation can lead to cellular apoptosis, inflammation, and fibrosis [158].

In lung tissues from IPF patients and bleomycin-induced mouse models, ER stress markers (p-eIF2α, p-IRE1α, ATF6) were significantly elevated [159]. Fibroblasts exposed to ER stress inducers become more susceptible to TGFβ-induced FMT [152,160]. Knockdown of the ER chaperone calreticulin in rat and human IPF fibroblasts reduces TGFβ1induced FMT and ECM production [161]. Overexpression of the ER stress-responsive C/EBP homologous protein (CHOP) enhances TGFβ1-induced FMT of lung resident mesenchymal/stromal cells by upregulating the TGFβ/SMAD signaling pathway [162]. Silencing PERK with siRNA or inhibiting it with a PERK inhibitor reduces the expression of differentiation markers, such as α-SMA and collagen IV, in lung fibroblasts [163]. Similarly, the loss of PGC-1α expression in fibroblasts promotes ER stress and supports the development of pulmonary fibrosis in a bleomycin-induced mouse model [159]. Furthermore, single-cell RNA sequencing data analysis revealed that fibroblast populations from the lungs of IPF patients have increased expression of mTOR metabolic targets and ATF4 which are key regulators of metabolic reprogramming in TGFβ-induced myofibroblasts [164]. Collectively, these findings indicate that ER stress significantly impacts the differentiation of lung myofibroblasts and contributes to the progression of pulmonary fibrosis (Figure 2).

## 5. Epigenetic Regulation of Myofibroblast Transformation

### Non-coding RNA:

Non-coding RNAs (ncRNAs) are a diverse group of RNA molecules that do not encode proteins but play crucial roles in regulating gene expression at various levels, including chromatin remodeling, transcription, post-transcriptional modification, and signal transduction [157]. ncRNAs are emerging as key regulatory molecules that influence gene expression and cellular functions, playing pivotal roles in various pathological conditions, including IPF. Recent studies have highlighted the significant role of ncRNAs, particularly microRNAs (miRNAs), long non-coding RNAs (lncRNAs), and circular RNAs (circRNAs), in the regulation of lung FMT and the progression of pulmonary fibrosis. These ncRNAs modulate this process through various mechanisms, including acting as competing endogenous RNAs (ceRNAs), sponging miRNAs, and interacting with key signaling pathways [165–170].

miRNAs play a pivotal role in regulating TGFβ1-induced FMT in pulmonary fibrosis. miRNAs such as miR-375 [171], miR-21 [172,173], miR-27a-3p [174], and miR-338–3p [165] have been extensively studied for their regulatory functions in this context. For example, miR-375 has been shown to prevent TGFβ1-induced transformation of lung fibroblasts into myofibroblasts by inhibiting the mitogen-activated protein kinase kinase 6 (MAP2K6)/P38 pathway, reducing collagen synthesis and myofibroblast markers such as α-SMA and periostin [171]. Conversely, miR-21 promotes fibroblast activation and differentiation via the glycolytic pathway [173]. Methyltransferase-like 3 (METTL3)-mediated m6A RNA methylation enhances the maturation of miR-21, promoting myofibroblast differentiation in TGFβ1-stimulated LR-MSCs [43]. Anti-miR-21 delivered by cationic liposomes inhibits myofibroblast differentiation and slows the progression of pulmonary fibrosis [175]. Additionally, miR-27a-3p acts as a negative regulator of lung fibrosis by targeting myofibroblast phenotypic markers, including α-SMA and the SMAD transcription factors SMAD2 and SMAD4, making it a potential therapeutic target to mitigate fibrotic progression [174]. Similarly, miR-338–3p blocks TGFβ-induced FMT through the induction of PTEN, further illustrating the diverse mechanisms by which miRNAs modulate TGFβ1 signaling and fibroblast activation [165].

Several lncRNAs have been identified as key regulators of FMT in pulmonary fibrosis. For instance, lncRNA CTD-2528L19.6 has been shown to prevent IPF progression by alleviating fibroblast differentiation through interactions with specific miRNAs, thereby modulating the expression of fibrosis-related genes [176]. Another lncRNA, ZFAS1, promotes the lung FMT and ferroptosis by acting as a ceRNA through miR-150–5p, highlighting the diverse mechanisms by which lncRNAs influence fibroblast behavior and fibrosis progression [177]. LINC00941, also known as lncIAPF, has been shown to promote FMT by forming an RNA-protein complex with ELAVL1 (HuR) to inhibit autophagy, a protective process in the lung. By blocking autophagy, LINC00941 enhances the stability of pro-fibrotic mRNAs [178].

CircRNAs, another class of ncRNAs, also play significant roles in lung fibroblast transformation and fibrosis. For example, circ0044226 has been found to regulate FMT and fibrosis by sponging miR-7 and regulating the expression of Sp1, a transcription factor involved in TGFβ1 signaling. This circRNA-mediated regulation underscores the complexity of ncRNA interactions and their impact on fibroblast activation [179]. Additionally, circRNAs such as CDR1as and circHIPK3 have been implicated in modulating fibrosis-related pathways, further emphasizing the potential of ncRNAs as therapeutic targets in pulmonary fibrosis [170].

### DNA methylation:

**Figure 3.**
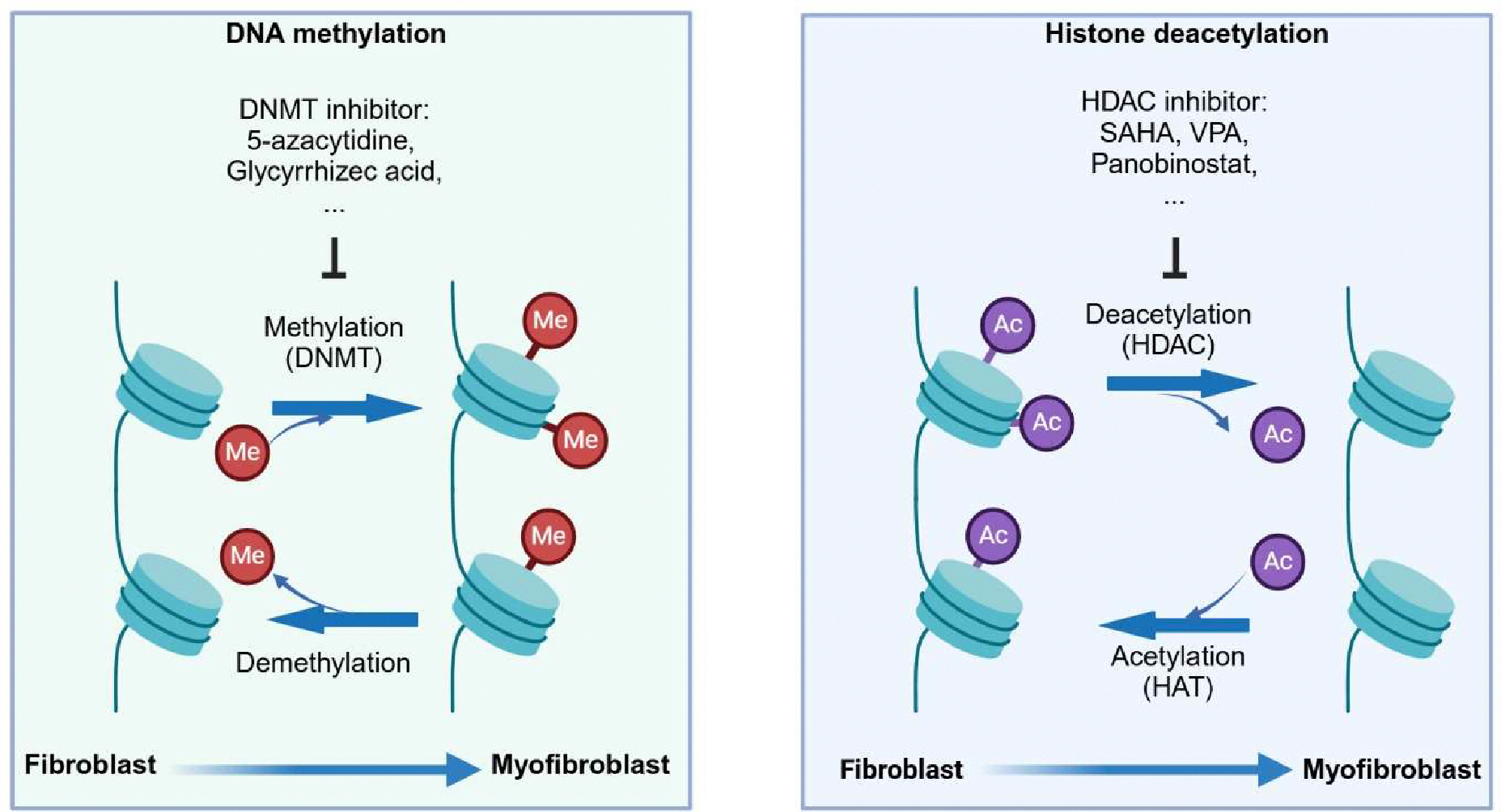
DNA methylation and histone deacetylation regulate myofibroblast transformation and serve as therapeutic targets. DNA methylation promotes FMT. DNA demethylation and DNMT inhibitor 5-azacytidine and glycyrrhizec acid inhibit FMT. Histone acetylation and HDAC inhibitor SAHA (suberoylanilide hydroxamic acid), VPA (valproic acid) and panobinostat suppress FMT.

Fibroblasts or myofibroblasts from IPF exhibit abnormal DNA methylation patterns (Figure 3). A study by Lee et al. identified 4251 hypomethylated sites across 1731 genes and 1599 hypermethylated sites across 725 genes in IPF fibroblasts compared to controls [180]. Another study confirmed significant DNA methylation differences in genes such as cyclin dependent kinase inhibitor 2B (CDKN2B), caspase recruitment domain protein 10 (CARD10), and O-6-methylguanine-DNA methyltransferase (MGMT) using bisulfite sequencing. These methylation changes corresponded to alterations in gene expression at both the mRNA and protein levels [181]. Specifically, hypermethylation at the CDKN2B gene locus in IPF fibroblasts leads to decreased CDKN2B expression, particularly in advanced fibrosis. This reduction enhances myofibroblast transformation rather than increasing fibroblast proliferation and is associated with increased serum response factor and myocardin-related transcription factor A, contributing to greater fibrosis severity in both human IPF and murine pulmonary fibrosis models [182]. Similarly, reduced expression of Thy-1 (CD90) in myofibroblasts within fibroblastic foci during pulmonary fibrosis is linked to hypermethylation of the Thy-1 promoter. TGFβ1 induces Thy-1 promoter methylation, and silencing DNA methyltransferase 1 (DNMT1) attenuates TGFβ1-induced DNMT activity, thereby preventing the downregulation of Thy-1 mRNA and protein expression and inhibiting TGFβ1-induced lung FMT [183].

Altered DNA methylation in IPF lung tissue and bleomycin-induced pulmonary fibrosis is associated with high expression of methyl-CpG-binding domain 2 (MBD2), a protein that decodes DNA methylation information [184]. MBD2 selectively binds to methylated CpG sites in the Edr1 promoter, suppressing its expression, thereby enhancing TGFβ/SMAD signaling, promoting fibroblast differentiation into myofibroblasts, and exacerbating pulmonary fibrosis [184]. Another well-known epigenetic reader, methyl-CPG-binding protein 2 (MECP2) has also been shown to facilitate TGFβ1-induced FMT by regulating α-SMA expression [185].

### Histone deacetylation:

Histone deacetylases (HDACs) are abnormally expressed in IPF tissues compared to non-diseased controls. Specifically, class I HDACs (HDAC1, HDAC2, HDAC3, HDAC8) and class II HDACs (HDAC4, HDAC5, HDAC7, HDAC9) are significantly elevated in IPF lung tissue [186]. In the lungs of IPF patients, nearly all HDACs are strongly induced in myofibroblasts within fibroblastic foci and in abnormal bronchial basal cells at sites of aberrant epithelialization [186]. The overexpression of these HDACs may contribute to the apoptosis-resistant phenotype of lung myofibroblasts, potentially by inhibiting FAS expression [186,187]. HDAC inhibition with suberoylanilide hydroxamic acid (SAHA) induces apoptosis in pulmonary fibrosis-derived myofibroblasts, at least partly mediated by upregulation of the pro-apoptotic gene Bak and downregulation of the anti-apoptotic gene B-cell lymphoma-extra large (Bcl-xL) [188].

HDACs play a crucial role in regulating FMT in pulmonary fibrosis (Figure 3). TGFβ-mediated FMT involves HDAC-dependent transcriptional repression. HDAC7 has been identified as a key factor in TGFβ-mediated FMT. Knockdown and inhibition of HDAC7 significantly reduce the expression of fibroblast activation genes such as NADPH oxidase 4 (NOX4), CTGF, and ACTA2 [189]. HDAC inhibitors like pracinostat attenuate TGFβ-mediated FMT and promote fibroblast quiescence by reversing epigenetic repression [189]. Furthermore, the inhibition of HDAC8 has been found to repress TGFβ1-induced FMT and ameliorate pulmonary fibrosis in vivo [190]. HDAC8 inhibition increases the expression of antifibrotic molecules such as PPARγ, highlighting its potential as a therapeutic target for treating IPF [190].

## 6. Regulation of Myofibroblast Transformation by Cell-Cell Interaction

### Epithelial-fibroblast interaction:

**Figure 4.**
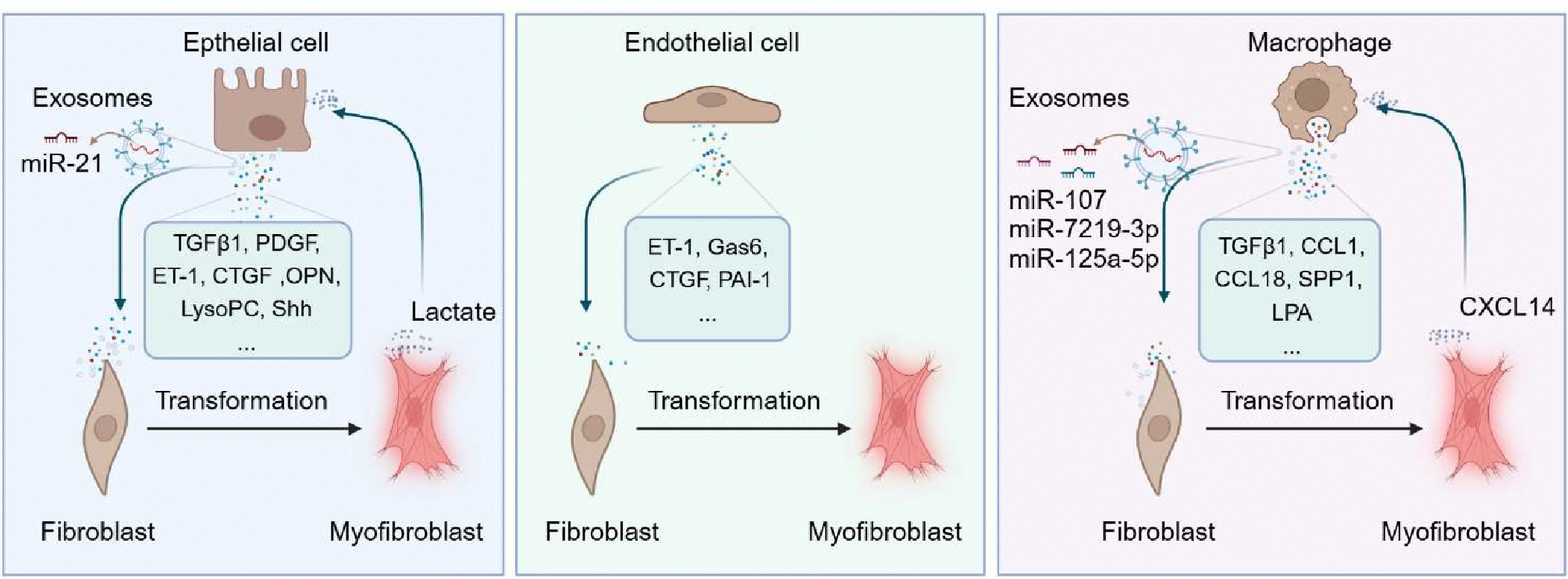
Cell-cell interactions regulate myofibroblast transformation. Lung epithelial cells, endothelial cells and macrophages help promote FMT though releasing cytokines and mediators. Myofibroblasts produce lactate, hydrogen peroxide and CXCL14, influencing the behavior of lung epithelial cells, endothelial cells and macrophages.

In the context of pulmonary fibrosis, damaged or hyperactivated lung epithelial cells produce various cytokines and metabolites including TGFβ1 [191,192], platelet-derived growth factor (PDGF) [193], endothelin-1 (ET-1) [194,195], CTGF [196,197], osteopontin (OPN) [198,199], and lysophosphatidylcholine (LysoPC) [200], Sonic Hedgehog (Shh) [201], as well as cargo-loaded exosomes [202], which contribute to the formation of lung myofibroblasts (Figure 4). Chronic cigarette smoke exposure in mice induces lung injury and pulmonary fibrosis. Cigarette smoke extract-treated bronchial epithelial cells secrete exosomes loaded with miR-21, which act on lung fibroblasts, reducing SMAD7 levels and activating the TGFβ1-SMAD3 pathway in FMT [202]. Similarly, arsenite-treated epithelial cells transfer EVs-encapsulated miR-21 to lung fibroblasts, reducing PTEN levels and phosphorylating AKT, which promotes glucose uptake and lactate secretion by lung fibroblasts, leading to myofibroblast differentiation [202].

Vice versa, myofibroblasts secrete several mediators, which influence the behavior of lung epithelial cells. For example, myofibroblasts release lactate which can upregulate global lactylation (Kla) and H3K18la levels in epithelial cells [203]. Hydrogen peroxide secreted by myofibroblasts acts as a death-inducing signal for alveolar epithelial cells, creating a cycle of extensive crosstalk between epithelial cells and fibroblasts or myofibroblasts [204].

### Endothelial-fibroblast interaction:

ECs release several key factors that significantly influence fibroblast activation and transformation. For example, endothelin-1 (ET-1), an endothelium-derived soluble peptide, is elevated in the lungs of IPF patients and in mouse models of pulmonary fibrosis (Figure 4). ET-1 directly induces the expression of matrix and matrix-associated genes via the MEK/ERK MAP kinase pathway [205]. Additionally, ET-1 promotes fibroblast resistance to apoptosis through the p38 MAPK and PI3K/AKT signaling pathways, suggesting that ET-1 acts as a paracrine regulator in the crosstalk between ECs and fibroblasts, thereby contributing to lung FMT [206]. Pulmonary microvascular endothelial cells (PMVECs) exposed to volatile organic compounds (VOCs) secrete growth arrest-specific 6 (Gas6), which binds to the Axl receptor on fibroblasts, inducing transformation [207].

In acute lung injury, CXCR7 in PMVECs supports airway epithelial cell proliferation and prevents fibrosis. However, chronic injury suppresses CXCR7 expression and recruits vascular endothelial growth factor receptor 1 (VEGFR1)-expressing perivascular macrophages [208]. This recruitment stimulates Wnt/β-catenin-dependent persistent upregulation of the Notch ligand Jagged1 in PMVECs, which, in turn, triggers Notch activation in perivascular fibroblasts, promoting FMT and pulmonary fibrosis [208].

Lung endothelial cells secrete ETS-related gene (ERG) which coordinates pulmonary angiogenic responses and regulates lung FMT in a paracrine manner [209]. Conditioned medium from ETS-related gene (ERG)-silenced ECs significantly promotes fibroblast activation and enhances the fibrogenic effects of TGFβ1 [209]. ERG-silenced ECs also secrete numerous fibrogenic mediators, including CTGF, Collagen Iα1, and PAI-1, while reducing the levels of basement membrane proteins such as collagen IV and laminin IV.

### Macrophage-fibroblast interaction:

In pulmonary fibrosis, macrophage-produced metabolites and soluble paracrine factors are critical mediators of the biological interplay between macrophage polarization and FMT (Figure 4). Depending on environmental cues, macrophages can polarize into either classically activated (M1) or alternatively activated (M2) phenotypes [210]. M2 macrophages are particularly associated with the secretion of profibrotic factors, notably TGFβ, which drives interstitial fibrosis deposition [211]. These M2 macrophages also secrete high levels of Wnt7a, promoting the differentiation of LR-MSCs into myofibroblasts and advancing pulmonary fibrosis through the activation of the Wnt/β-catenin signaling pathway [212]. Conditioned medium from interstitial macrophages sorted at day 7 post-bleomycin treatment induces myofibroblast differentiation in vitro [213].

CCL1 secreted by alveolar macrophages and CD4+ T cells recruits and activates lung fibroblasts by binding to autocrine motility factor receptor (AMFR) and activating the ERK-p70S6K cascade [17]. Alveolar macrophages in IPF patients produce abundant CCL18 [214], which stimulates collagen production and lung fibroblast transformation via Sp1 signaling and basal SMAD3 activity [215]. Moreover, native collagen induces CCL18 expression in normal human alveolar macrophages, suggesting a positive feedback loop between AMs and fibroblasts [214]. TGFβ1-stimulated lung fibroblasts secrete CXCL14, which recruits bone marrow-derived macrophages (BMDMs) and favors CXCL14-mediated M2 polarization of macrophages in crystalline silica-induced pulmonary fibrosis [216].

Macrophage-derived exosomes are rich in miRNAs that modulate gene expression in recipient fibroblasts and induce the transformation of lung fibroblasts into myofibroblasts. For instance, miR-107 [217], miR-7219–3p [218], and miR-125a-5p [219,220] in macrophage-derived exosomes have been implicated in regulating fibroblast transformation and ECM production. MiR-7219–3p promotes myofibroblast differentiation by targeting spouty1 (SPRY1) through the ERK/MAPK signaling pathway [218]. Macrophage-derived exosomes can also carry cytokines, such as SPP1 which promotes the migration, adhesion, and proliferation of fibroblasts and enhances TGFβ1-induced FMT [198].

Single-cell RNA sequencing studies have identified several distinct subsets of macrophages with specific pro-fibrotic roles in pulmonary fibrosis [221]. One such subset, characterized by high expression of phospholipase A2 group VII (PLA2G7), secretes excessive lysophosphatidic acid (LPA), which binds to lysophosphatidic acid receptor-2 (LPA2) on the surface of fibroblasts. This interaction promotes the transformation of fibroblasts into myofibroblasts, contributing to fibrosis [222]. Another pro-fibrotic macrophage subset is CX3C motif chemokine receptor 1 (CX3CR1) ^+^SiglecF^+^ macrophages [223]. This subset of macrophages was found to be a source of PDGF-aa within the fibrotic niche, which facilitates fibroblast migration and proliferation. On the other hand, lactate from myofibroblasts can induce histone lactylation in macrophages, leading to a profibrotic phenotype [224]. Thus, paracrine interactions between macrophages and fibroblasts help maintain fibroblastic properties and drive pulmonary fibrosis [223].

## 7. Therapeutic Potential on Myofibroblast Transformation

### Targeting signaling pathways of myofibroblast transformation:

FMT is driven by several key signaling pathways, making these pathways potential targets for therapeutic intervention. Central to the therapeutic potential is the inhibition of the TGFβ signaling pathway [95,225]. Therapeutic agents such as nintedanib and pirfenidone, which inhibit TGFβ signaling and other fibrotic pathways, have become the preferred options for treating IPF, as they help slow down the progression of the disease [226,227]. Additionally, several compounds that inhibit lung FMT by directly or indirectly targeting the TGFβ1 signaling pathway have shown strong anti-fibrotic effects [22,127,228].

Targeting the MAPK pathway has also demonstrated anti-fibrotic effects in preclinical studies, with evidence showing that it inhibits lung FMT in vivo [25,81,117]. For example, the p38α-specific inhibitor VX-702 attenuated bleomycin-induced fibrosis and promoted fibrosis regression in mice [81]. MAPK inhibitor SB203580 and PD98059 have been shown to inhibit vincristine- and amiodarone-induced activation of lung fibroblasts and FMT [25,117]. Beyond TGFβ and MAPK, modulating the Wnt/β-catenin signaling pathways—each implicated in FMT and fibrosis—offers additional therapeutic avenues [133,229].

In aging and IPF, mTORC1 activation reduces autophagy, leading to apoptosis resistance and acceleration of the senescence of airway epithelial cells and the differentiation of lung myofibroblasts [230]. Restoring autophagy could be beneficial for treating pulmonary fibrosis [148,231]. Aspirin has been shown to alleviate pulmonary fibrosis in bleomycin-induced models and FMT via the PI3K/AKT/mTOR-mediated autophagy pathway [232]. Similarly, isoliquiritigenin, a flavonoid compound, has been shown to inhibit TGFβ1-induced fibrogenesis by activating autophagy in MRC-5 cells through suppression of the PI3K/AKT/mTOR pathway [233]. FGF21 significantly inhibits the activation of the AKT signaling pathway, reducing TGFβ-induced FMT and preventing uncontrolled fibroblast proliferation [234]. Regulating autophagy in lung fibroblast activation presents a promising avenue for therapeutic intervention in pulmonary fibrosis.

### Targeting DNA methylation:

5-aza-2′-deoxycytidine (5-aza) is a cytidine analog that inhibits the enzymatic activity of DNA methyltransferases (DNMTs) by forming a covalent bond, thereby reducing DNA methylation levels in cells [235]. Treatment with 5-aza restores Thy-1 mRNA expression and inhibits α-SMA expression and FMT [236]. Additionally, 5-aza attenuates the invasion and migration of IPF lung fibroblasts by reducing BMP endothelial cell precursor-derived regulator (BMPER) expression [237]. Furthermore, 5-aza and glycyrrhizic acid (GA) demethylate the PPARγ promoter, restoring TGFβ1-induced PPARγ deficiency in lung fibroblasts and alleviating pathological changes in fibrotic lung tissue *in vivo* [238].

### Targeting histone deacetylation:

The Fas promoter in fibroblasts from bleomycin-treated mice shows reduced histone acetylation, contributing to low Fas expression and increased expression of HDAC2 and HDAC4 [187]. Treatment of lung fibroblasts from bleomycin-induced fibrosis with trichostatin A or SAHA induces cell surface Fas expression and restores sensitivity to Fas-induced apoptosis [187]. SAHA induces apoptosis in IPF myofibroblasts, partly mediated by upregulation of the pro-apoptotic gene Bak and downregulation of the anti-apoptotic gene Bcl-xL [188]. HDAC inhibitors such as panobinostat (LBH589) and valproic acid (VPA) have been shown to alleviate the anti-apoptotic phenotype of IPF fibroblasts by regulating anti-apoptotic genes and ER stress [186]. A study comparing the antifibrotic effects of the pan-HDAC inhibitor panobinostat and pirfenidone on IPF fibroblasts concluded that panobinostat induces cell cycle arrest and apoptosis, suggesting it is more effective than pirfenidone in inactivating IPF fibroblasts [239].

HDAC inhibitors like pracinostat have shown potential in attenuating TGFβ-induced FMT by reversing histone deacetylation, thereby preventing the repression of antifibrotic genes [189]. Pracinostat treatment in lung fibroblasts results in increases in histone acetylation at the promoter regions of antifibrotic genes like peroxisome proliferator-activated receptor γ co-activator-1α (PGC-1α), Rho GTPase-activating protein 12 (ARHGAP12), and superoxide dismutase 2 (SOD2) leading to inhibitions of FMT and TGFβ-induced expression of α-SMA and collagen, thereby mitigating pulmonary fibrosis [189].

Selective HDAC inhibitors targeting specific isoforms, such as HDAC6 and HDAC8, have demonstrated efficacy in reducing fibrosis through modulating FMT and ECM production [240,241]. The development of selective HDAC inhibitors may provide more targeted therapeutic options, minimizing side effects and enhancing treatment efficacy. Further clinical studies are warranted to validate their efficacy and safety in patients with IPF and other fibrotic lung diseases.

## 8. Conclusions and Perspectives

Single-cell transcriptomic analysis has revealed significant heterogeneity among pulmonary fibroblasts, which originate from diverse cell types and exhibit the ability to adapt to an altered microenvironment during the repair of lung injury under pathological conditions [15]. The transformation of pulmonary fibroblasts into myofibroblasts is a central process in the progression of pulmonary fibrosis, driven by intricate signaling pathways and influenced by key interactions with epithelial cells, endothelial cells, and immune cells. Although considerable progress has been made in understanding the cellular and molecular mechanisms as well as the differentiation trajectories involved, effectively targeting these pathways to prevent or reverse fibrosis remains a significant challenge.

The plasticity of myofibroblasts presents substantial therapeutic potential. During the recovery phase of pulmonary fibrosis, myofibroblasts can transdifferentiate into lipofibroblasts, a process that involves the reversal of fibrotic states [37,84]. This transdifferentiation is influenced by the evolving microenvironment, which includes signals that promote lipid storage and metabolic changes within cells. A deeper understanding of the molecular mechanisms driving this shift could provide new avenues for targeted therapies aimed at reversing fibrosis. Future research should continue to explore the plasticity of myofibroblasts, identify novel therapeutic targets, and refine strategies for regulating cell-to-cell interactions.

Interplay between genetics and epigenetics may determine the genetic variants associated with susceptibility to lung fibrosis and their associated therapeutic response [242]. Epigenetic modifications and gene expression are highly specific to cell types, and their relationship with IPF remains complex. Advances in single-cell epigenomics and spatial transcriptomics have been instrumental in elucidating the heterogeneity of cell populations within the lung microenvironment [93]. However, epigenomic studies still require more sophisticated methodologies to achieve cell-type-specific analysis. By characterizing the epigenetic landscapes of individual cell types and their spatial organization within the lung microenvironment, it is possible to identify and interpret the contributions of cell-specific mechanisms to disease progression, as well as to pinpoint potential drug targets for therapeutic intervention [91].

HDACs are emerging as potential molecular targets for the treatment of pulmonary fibrosis. Growing evidence suggests that HDAC inhibitors exhibit anti-fibrotic effects in bleomycin-induced pulmonary fibrosis animal models, and in vitro studies demonstrate that HDAC inhibitors suppress the formation of myofibroblasts and promote their apoptosis [243–245]. However, their clinical application in fibrotic diseases has yet to be approved. Furthermore, research and development of selective HDAC inhibitors remain in the early stages. Therefore, assessing the efficacy and selectivity of HDAC inhibitors and modifying lead compounds to generate highly selective and potent inhibitors will be crucial areas for future investigation.
